# “Zero Tolerance” for disruptive health work behaviors

**DOI:** 10.31744/einstein_journal/2019CE5306

**Published:** 2019-10-03

**Authors:** Alexsandro Tartaglia

**Affiliations:** 1 Escola Bahiana de Medicina e Saúde Pública SalvadorBA Brazil Escola Bahiana de Medicina e Saúde Pública, Salvador, BA, Brazil; Secretaria da Saúde do Estado da Bahia, Salvador, BA, Brazil; Hospital Geral Roberto Santos, Salvador, BA, Brazil.; Secretaria da Saúde do Estado da Bahia SalvadorBA Brazil; Hospital Geral Roberto Santos SalvadorBA Brazil

Letter to the editor,

The healthcare work process is complex and engages several health professionals, who along with other professionals develop multiple activities essential to support the organizational structure. Thus, continuous interaction among players is required to carry out patient care. Any teamwork is challenging, for there is a diversity of individuals and ideas in face of a work process shared by all.^1^

Unfortunately, a major problem affecting personal and financial costs in healthcare services has being ignored for a long time: disruptive behaviors of healthcare workers. The stressful setting of health organizations is fertile ground for triggering disruptive and uncivil behaviors. These behaviors have been studied in management and social psychology literature during the past two decades, and are being acknowledged as a growing threat to productivity in the workplace, workers’ motivation, absenteeism, staff loyalty to the organization, and to physical and emotional wellbeing.^2^ Disruptive behaviors specific to healthcare studies include some actions, such as shouting or talking loudly, disrespectful interaction, use of abusive language, being admonished in front of colleagues and patients, suffering insults and experiencing abusive anger. Individuals usually exhibiting these behaviors have been described in studies by several expressions, as toxic, uncivil, disruptive and intimidating.^2 - 4^

In a study with over 400 leaders, more than 94% had experienced dealing with a toxic person in the workplace.^2^ In another study, 25% of healthcare workers believed that disruptive behaviors were positively correlated to patient mortality, and 49% stated that staff intimidation resulted in drug administration errors.^2^ In 2004, the American College of Physician Executives reported that 80% of physicians showed disrespectful behavior toward their team. Although considerable attention has been given to physicians` role as primary instigators of disruptive behaviors, they are not the only culprits. Researchers have shown abusive behavior among health professionals.^2^ Felblinger^5^ reported that nurse against nurse horizontal violence is second only to occurrences practiced by physicians – the truth is no one is immune!

Throughout the years, health organizations have nourished disrespectful behavior, ignoring it, and thus, tacitly accepting it. As a result, by considering the behavior as a normal style of communication, the healthcare culture has allowed for a certain degree of disrespect.^2 , 3^ The proliferation of toxic behaviors is highly influenced by the culture of an organization. There is a call for an urgent change from a paternalist culture, dominated by medical doctors, to a team-based approach, in which all members are accountable.^2 - 5^

Accordingly, in 2008, the Joint Commission established that, from January 1st, 2009, every hospital organization should introduce policies and procedures to deal with staff showing disrespectful and inappropriate behaviors in the workplace. Such action is a warning to healthcare practices, aiming to design and implement healthcare settings with respectful engagement and “zero tolerance” to disruptive, uncivil and intimidating behaviors of any professional.^6^

Pearson et al.,^7^ found that ill behaviors caused the following effects in organizations: 12% of victims of toxic individuals quit their jobs; 48% lower work effort; 47% reduce work schedule; 38% decrease quality of work; 68% reported worsening performance; 80% reported wasted time worrying about the unpleasant situations that occurred at work; 63% wasted time avoiding the person who presented disruptive behavior, and 78% stated that organizational loyalty diminished.

Given the above, it is evident that safety consequences are implicated in this context, thus compromising patient care. In a hostile environment, communication is impaired and can have a direct impact on patient care outcomes.^3 , 4^ Disruptive behaviors have been associated with adverse events, impaired patient safety and even patient mortality. Additionally, disruptive behaviors are at the root of the difficulties met in developing team-based approaches to improve care. Such effects may impair clinical judgment as professional performance is affected.^4 , 7^ If confidence and capability decrease as a result of toxic behavior, consequently the quality of patient healthcare and outcomes are negatively affected.

The toxic effects of incivility in health culture have only been revealed recently. To change from a “toxic culture” to a “respect culture”, an approach of the complete system must be implemented. Therefore, any effective intervention plan must start with the approach to the whole system to fight the problem, and not only by means of admonition and punishment, that have been so typical when dealing with this scenario. Leaders are accountable for dealing with the problem, increasing awareness on the topic and encouraging others to change behaviors.^2^ Then, it´s up to leadership to act...

Zero Tolerance to disruptive behaviors is beneficial to health organizations, teams, individuals (professional and social being) and to patients. It is necessary to acknowledge this reality, fight these ill behaviors and repeal silent approaches. Thus, the more active, analytic and reflexive the process, the higher the chances for us to make real changes.

## References

[1] 1. Cecilio LC, Lacaz FA. Temas fundamentais da reforma sanitária. O trabalho em saúde. Cidadania para a saúde [Internet]. Rio de Janeiro: Cebes; 2012 [citado 2019 Maio 10]. Disponível em: http://cebes.org.br/site/wp-content/uploads/2015/02/7O-Trabalho-em-Sa%C3%BAde.pdf

[2] 2. Holloway EL, Kusy ME. Disruptive and toxic behaviors in healthcare: zero tolerance, the bottom line, and what to do about it. J Med Pract Manage. 2010;25(6):335-40. 20695243

[3] 3. Grissinger M. Unresolved disrespectful behavior in health care: practitioners speak up (again)-part 1. P T. 2017;42(1):4-23. PMC5215268 28090154

[4] 4. American Nurses Association. Position Statement. Incivility, Bullying, and Workplace Violence [Internet]. Maryland: (USA); 2015 [cited 2019 June 25]. Available from: https://www.nursingworld.org/practice-policy/nursing-excellence/official-position-statements/id/incivility-bullying-and-workplace-violence/

[5] 5. Felblinger DM. Bullying, incivility, and disruptive behaviors in the healthcare setting: Identification, impact, and intervention. Front Health Serv Manage. 2009;25(4):13-23. 19603687

[6] 6. The Joint Comission. Sentinel Event Alert. Behaviors that undermine a culture of safety. Sentinel Event Alert. 2008;9(40):1-3. 18686330

[7] 7. Pearson C, Porath CL. On the nature, consequences and remedies of workplace incivility: No time for “nice”? Think again. Academy of Management Executive. 2005;19(1):7-18.

