# Supplementary material for: “Zero Tolerance” for disruptive health work behaviors
Source: Einstein (Sao Paulo). 2019 Oct 3;17(4):eCE5306. doi: 10.31744/einstein_journal/2019CE5306 (PMC6777647; doi:10.31744/einstein_journal/2019CE5306)
Supplement: Translation

# Translation: “Tolerância Zero” para comportamentos disruptivos no trabalho em saúde

**Authors:** Alexsandro Tartaglia

**Affiliations:** 1 Brasil Escola Bahiana de Medicina e Saúde Pública, Salvador, BA, Brasil; Secretaria da Saúde do Estado da Bahia, Salvador, BA, Brasil; Hospital Geral Roberto Santos, Salvador, BA, Brasil.

Carta ao editor,

O processo de trabalho em saúde é complexo, envolvendo diversos profissionais de saúde e outros grupos de trabalhadores, que desenvolvem uma série de atividades necessárias para a manutenção da estrutura institucional. Dessa forma, exige-se uma interação contínua entre os atores, que originam as atividades para atender os usuários. Todo trabalho em equipe constitui um desafio, porque há uma diversidade de pessoas e ideias diante de um processo de trabalho comum a todos.^1^

Infelizmente, um problema significativo e que afeta os custos pessoais e financeiros nos serviços de saúde tem sido ignorado há muito tempo: comportamentos disruptivos, por parte dos profissionais de saúde. O ambiente estressante das organizações de saúde é um terreno fértil para desencadear comportamentos disruptivos e incivilizados. Esses tipos de comportamentos têm sido estudados na literatura de gestão e psicologia social nas últimas duas décadas e reconhecidos como ameaça crescente à produtividade no local de trabalho, à motivação do trabalhador, ao absenteísmo, à fidelização do trabalhador na instituição e ao bem-estar, tanto físico quanto emocional.^2^ Específicos para pesquisas em saúde, os comportamentos disruptivos incluem itens como gritar ou levantar a voz, interagir de forma desrespeitosa, utilizar linguagem abusiva, ser repreendido na frente de colegas e pacientes, sofrer insultos e passar por raiva abusiva. As pessoas que habitualmente exibem esses comportamentos foram mencionadas de vários modos, como tóxicas, incivis, disruptivas e intimidantes.^2 - 4^

Pesquisa com mais de 400 líderes revelou que 94% deles tiveram que lidar com uma pessoa tóxica no trabalho.^2^ Em outro estudo, 25% dos profissionais de saúde acreditam que comportamentos disruptivos estão positivamente correlacionados com a mortalidade do paciente, e 49% afirmaram que a intimidação de outro profissional resultou em má administração de medicamentos.^2^ Em 2004, o *American College of Physician Executives* relatou que 80% dos médicos foram desrespeitosos com a equipe. Embora tenha havido considerável atenção ao papel dos médicos como instigadores primários de comportamentos disruptivos, eles não são os únicos culpados. Pesquisadores relatam abusos entre muitos profissionais de saúde.^2^ Felblinger^5^ relatou que a violência horizontal de enfermeiras contra enfermeiras perde apenas para eventos praticados por médicos – a verdade é que ninguém está imune!

Organizações de saúde têm alimentado o problema do comportamento desrespeitoso por anos, ignorando-o e, assim, tacitamente o aceitando. Dessa forma, a cultura de cuidado em saúde tem permitido certo grau de desrespeito, ao considerar que isso é um estilo normal de comunicação.^2 , 3^ A proliferação de comportamentos tóxicos é altamente influenciada pela cultura da organização. Deve haver uma mudança urgente de uma cultura paternalista, dominada por médicos, para uma abordagem baseada em equipe, na qual todos os membros são responsáveis.^2 - 5^

Nesse sentido, em 2008, a *Joint Commission* determinou que, a partir de 1º de janeiro de 2009, todas as organizações hospitalares deveriam ter políticas e procedimentos em vigor que abordassem comportamentos desrespeitosos e inadequados entre os funcionários. Tal ação é um alerta para as práticas em saúde, a fim de projetar e implementar estratégias para criar ambientes de cuidados de saúde de engajamento respeitoso, que tenham “tolerância zero” para comportamentos disruptivos, incivis e intimidadores advindos de qualquer profissional.^6^

Pearson et al.,^7^ descobriram que maus comportamentos têm os seguintes efeitos nas organizações: 12% das vítimas de pessoas tóxicas desistem do trabalho; 48% diminuem o esforço no trabalho; 47% reduzem o tempo de trabalho; 38% diminuem a qualidade do trabalho; 68% disseram que o desempenho decresceu; 80% relataram que perderam tempo se preocupando com situações desagradáveis ocorridas no trabalho; 63% perderam tempo evitando a pessoa que apresentou o comportamento disruptivo e 78% afirmaram que o comprometimento com a organização diminuiu.

Diante do exposto, fica evidente que consequências na segurança estão implicadas nesse contexto, comprometendo, assim, a assistência ao paciente. Em um ambiente hostil, a comunicação é prejudicada e pode ter impacto direto nos resultados dos cuidados aos pacientes.^3 , 4^ Comportamentos disruptivos têm sido associados a eventos adversos, ao comprometimento na segurança do paciente e, até mesmo, à mortalidade do paciente. Além disso, esses modos estão na raiz das dificuldades encontradas no desenvolvimento de abordagens baseadas em equipe para melhorar os cuidados. Tais efeitos podem prejudicar o julgamento clínico, na medida em que o desempenho do profissional é afetado.^4 , 7^ Se a confiança e a competência diminuírem como resultado de comportamentos tóxicos, consequentemente, a qualidade da assistência ao paciente e os resultados de cuidados são afetados negativamente.

Os efeitos tóxicos da incivilidade na cultura da saúde têm sido revelados apenas recentemente. Para que mudanças de “culturas de toxicidade” para “culturas de respeito” sejam criadas, uma abordagem completa do sistema deve ser implementada. Portanto, qualquer plano efetivo de intervenção deve começar com a abordagem de todo o sistema para combater o problema, e não simplesmente por meio de procedimentos de repreensão e punição, que têm sido típicos na abordagem dessa realidade. A responsabilidade por abordar o problema pertence aos líderes, que precisam aumentar a conscientização sobre o tema e inspirar outros a mudarem comportamentos.^2^ Cabe, então, à liderança agir...

Tolerância zero a comportamentos disruptivos faz bem às organizações de saúde, à equipe, ao indivíduo (profissional e ser social) e aos pacientes. É necessário reconhecer essa realidade, combater esse mal e repelir posições quietistas. Assim, quanto mais ativo, crítico e reflexivo for esse processo, maiores serão as chances de produzirmos mudanças reais.
